# The impact of childhood obesity on different fracture sites

**DOI:** 10.1038/s41598-025-11203-7

**Published:** 2025-07-20

**Authors:** Wentao Yang, Zhiyu Zhou, Wei Gu, Xu Wang, Lei Ni

**Affiliations:** 1 Department of Endocrinology, Children’s Hospital of Nanjing Medical University, Nanjing, 210008 China; 2 Department of Orthopedics, Children’s Hospital of Nanjing Medical University, Nanjing, 210008 China; 3 Clinical Medical Research Center, Children’s Hospital of Nanjing Medical University, Nanjing, 210008 China

**Keywords:** Obesity, Fracture, Fracture site, Orthopaedics, Paediatrics, Weight management

## Abstract

The prevalence of childhood obesity has been increasing worldwide, garnering increasing public attention due to various complications and long-term effects. Many researchers have indicated that obese children experienced higher fracture risk compared with nonobese children. However, the findings from different researchers reported a controversial result and few of them paid attention to the differences in BMI Z-score and obesity between different fracture sites, which would be quite useful since these can guide obese children in protecting specific regions. This study comprised 17,942 hospitalized children diagnosed with fractures and 3219 healthy children who came for physical examination and had not been diagnosed with any illness. Data on age, gender, fracture seasons, fracture sites, height, weight, total cholesterol, triacylglycerol, and high-density lipoprotein were collected. One-way ANOVA, t-test, Chi-square test, propensity score matching, and logistic regression models were used in statistical analysis. Children with lower limb fractures exhibited the highest BMI Z-score (0.58 ± 1.74), followed by those with upper limb fractures (0.50 ± 1.52), axial fractures (0.31 ± 1.56), and head fractures (-0.02 ± 1.52). In terms of obesity, children with lower limb fractures exhibited the highest obesity rate (20.20%), whereas those with upper limb fractures (15.61%) and axial fractures (14.96%) displayed comparable obesity rates. Children with head fractures had the lowest obesity rate (8.42%). Moreover, BMI Z-score (2.43 ± 1.22vs0.15 ± 1.43, P < 0.001), obesity (62.97%vs8.61%, P < 0.001) and dyslipidemia (31.22%vs24.67%, P < 0.001) were statistically significant difference between the fracture and healthy groups. The logistic regression models showed that BMI Z-score was associated with an increased risk of fracture (P < 0.001, OR = 4.89, 95%CI: 4.53–5.27). This study suggests that children with lower limb fractures exhibited the highest BMI Z-score and obesity rate, while those with head fractures had the lowest BMI Z-score and obesity rate. When compared with the healthy group, fracture children had higher BMI Z-score, obesity, and dyslipidemia rates. In addition, BMI Z-score was associated with an increased risk of fractures.

**Supplementary Information:**

The online version contains supplementary material available at 10.1038/s41598-025-11203-7.

## Introduction

Obesity, a common public health issue, has become increasingly prevalent globally. In 2020, approximately 22%of children aged 5–19 were overweight or obese worldwide, and 37% were reported in China^1^. Childhood obesity may cause various psychosocial, cardiovascular, endocrinological, pulmonary, and musculoskeletal complications, such as type 2 diabetes and hypertension^2^. Moreover, most childhood obesity may routinely translate into adult obesity, which makes it a serious public health problem worldwide^3^. To compound the problem, many research indicated that obese children would face higher fracture risk compared with nonobese children^4–6^. The increased fracture risk in obese children can be explained from multiple perspectives, including endocrine disorders, lipid metabolic disturbances, disruption of the bone microenvironment, and low-grade inflammation^7–10^.

Bone fracture is a quite common adverse event in childhood. A study conducted in the USA indicated that approximately half of children sustained a fracture during childhood, with almost a quarter of boys and 15% of girls suffering multiple fractures^11^. Childhood fractures would bring immediate painful experiences to children as well as a longtime social burden for families^12^. Furthermore, the history of prior fractures is one of the strongest predictors of future fractures^13^. A cohort study conducted in New Zealand indicated that childhood fracture history is associated with persistent skeletal fragility in adulthood^14^. Therefore, avoiding or reducing childhood fractures is of great significance.

Several researchers have reported that the fracture risks of obese children are greater than that of their nonobese counterparts at various fracture sites. For instance, a research in Italy found that the overweight/obesity rate increased in children with upper or lower limb fractures^4^. A study in Spain on preschool children also indicated that preschool children with an overweight or obese range BMI had an increased incidence of upper and lower limb fractures in childhood compared with contemporaries of normal weight^5^. Study in the USA indicated that obese children had a greater risk of pelvic bone fracture than their nonobese counterparts in sideways falls by using computational modeling methodology^6^. However, some research reports have conflicting opinions. Research in the USA indicated that no difference was observed in the risk of upper-extremity fracture within different BMI groups^15^. Most of the previous studies focused on the differences between obese and nonobese children in only one or two fracture sites. Few of them paid attention to the differences in BMI Z-score and obesity in various fracture sites. These suggested a need for a comprehensive study to investigate the impact of BMI Z-scores at various fracture sites.

Therefore, this study included fractures at various sites, such as axial (spine/thorax but also including pelvis and clavicle), upper limb (proximal upper limb, wrist/forearm, hand), lower limb (femur, tibia/fibula/ankle, and foot), and head (skull and facial bones) and aimed to accomplish two objectives. For one part, our study involves comparing within the fracture group to explore the relationship between obesity, BMI z-scores, and fracture sites. For the other part, we conducted an analysis between the fracture group and healthy controls to investigate the association of obesity, BMI z-scores, and blood lipid profiles with fracture risk.

## Methods

### Study design and study population

**Fig. 1:**
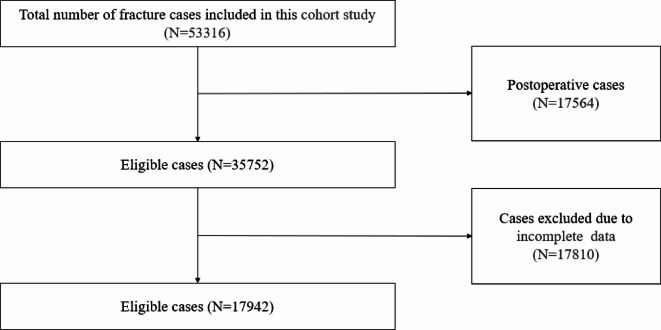
Flow chart of children included in the fracture group in this study.

From August 2016 to August 2024, we initially enrolled a total of 53,316 hospitalized children diagnosed with fractures at different sites by a pediatrician at the Children’s Hospital of Nanjing Medical University. Only children who were diagnosed with fractures for the first time were included as cases or fracture groups. Post-operative cases, subjects with incomplete data were excluded^16^. Finally, 17,942 eligible children were included in the fracture group in this study (Fig. 1).

**Fig. 2:**
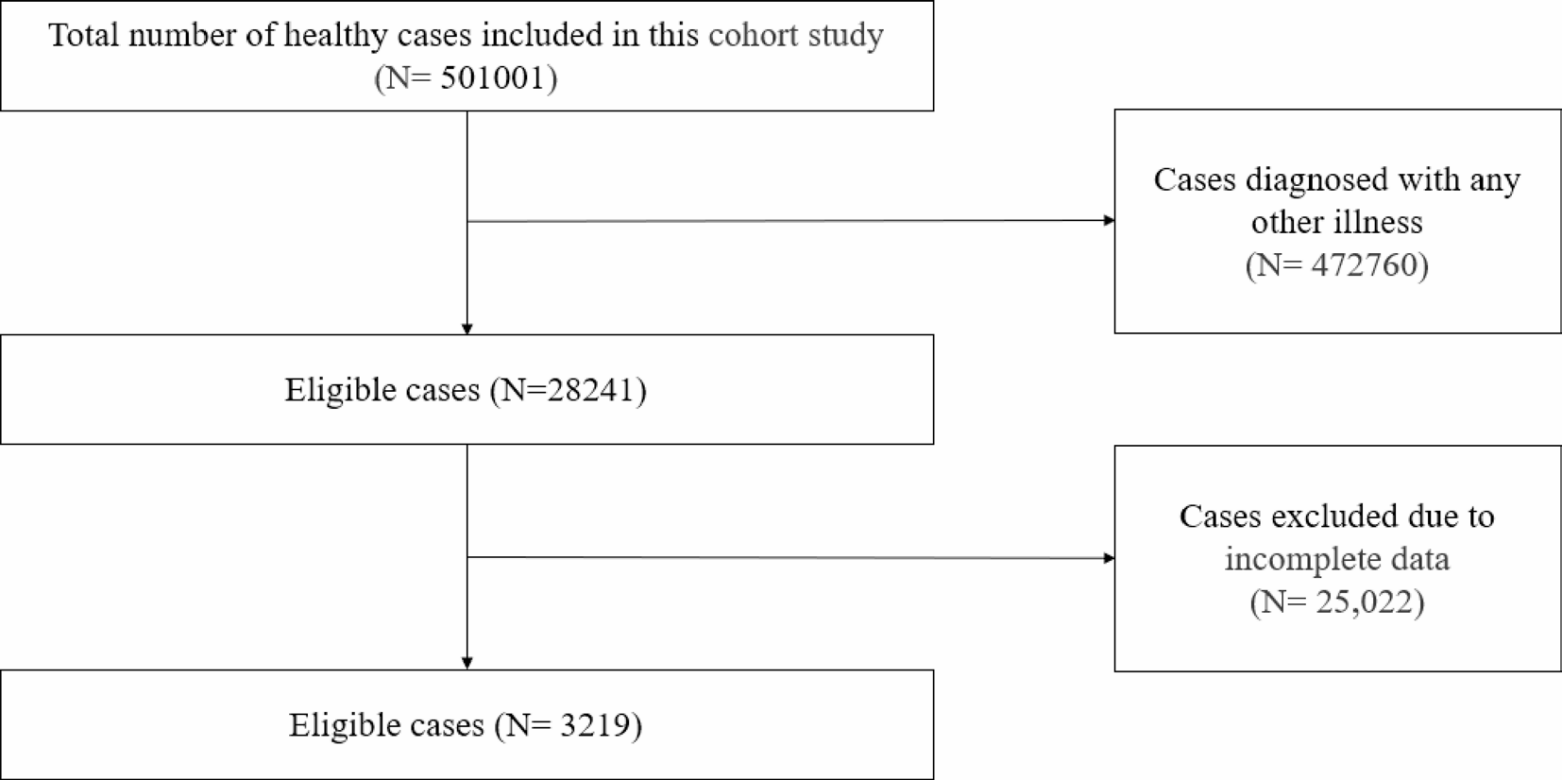
Flow chart of the cases included in the healthy group in this study.

As for the healthy group, we initially recruited a total of 501,001 children who visited the outpatient clinic of the Child Health Care department in the Children’s Hospital of Nanjing Medical University from June 2017 to September 2024. Only children who came for physical examination and had not been diagnosed with any illness were included as a healthy group. Subjects with incomplete data were excluded^16^. Finally, 3219 eligible children were included in the healthy group in this study (Fig. 2).

### Data collection

The information collected from the hospital records for the fracture and healthy groups were age, gender, height, weight, and lipid profile such as total cholesterol, triacylglycerol, and high-density lipoprotein. In this hospital, blood samples for lipid detection required patients to fast for at least 4 h before collection. High-density lipoprotein cholesterol was detected by the chemical precipitation method, while triacylglycerol and total cholesterol were detected by both chemical and enzymatic methods. The record about fracture seasons and fracture sites were only collected from the fracture group. Body mass index (BMI) was calculated by taking weight (in kg)/height^2^ (in m^2^). All BMI Z-scores were calculated by R package “anthro” and “anthroplus” following the World Health Organization (WHO) growth standard. BMI Z-score groups were defined as: underweight (BMI Z-score ≤−2), normal weight (−2 < BMI Z-score < 1), overweight (1 ≤ BMI Z-score ≤ 2), and obesity (BMI Z-score > 2). Fracture sites were defined as axial (spine/thorax but also including pelvis and clavicle), upper limb (proximal upper limb, wrist/forearm, hand), lower limb (femur, tibia/fibula/ankle, and foot), and head (skull and facial bones). Data used in this study were anonymous, and no individual identifiable information was available.

### Statistical analysis

Descriptive statistics were presented as mean ± standard deviation (SD) or count and percentage. The one-way analysis of variance (ANOVA) was used to compare differences in BMI Z-score between different fracture sites. The least significant difference (LSD) test was used as a post hoc test for results with statistically significant differences after One-way ANOVA. The chi-square test was used to compare the difference in BMI Z-score groups (obesity status) between different fracture sites. We performed propensity score matching at the ratio of 1:1 between the fracture and healthy groups according to age and gender. After the propensity score matching, we compared the differences between the fracture and healthy groups using the chi-square test for categorical data t-test for continuous data. Logistic regression models were used to estimate the associations between BMI Z-score and odds of fracture. These analyses were all performed with SPSS statistical software (Version 26.0. Armonk, NY: IBM Corp) and “anthro”, “anthroplus” and “MatchIt” packages in R (version 4.2.3).

Power analysis was performed for each statistical analysis. Based on the sample size obtained in our study, with the significance level set at 0.05 and using Cohen’s recommended medium effect size, the statistical power for all analyses was calculated to be 1. Thus, it is concluded that all statistical analyses had sufficient power.

## Result

A total of 17,942 individuals were included in the fracture group, including 11,738 (65.42%) males and 6204(34.52%) females. The mean age of the participants was 6.30 ± 3.76 years. The majority of the children 10,175(56.71%) sustained upper limb fractures. The more details are shown in Table 1.

**Table 1:** Distribution of children’s characteristics in the fracture group.

Variables (*N* = 17942)	Mean ± SD/*N*(%)
Age (year)	6.30 ± 3.76
Gender	
Male	11,738(65.42)
Female	6204(34.57)
Fracture season	
Spring	4965(27.67)
Summer	5337(29.75)
Autumn	4873(27.16)
Winter	2767(15.42)
Fracture site	
Upper limb	10,175(56.71)
Lower limb	3267(18.21)
Axial	842(4.69)
Head	3658(20.39)
BMI Z-score	0.40 ± 1.58
BMI Z-score group	
Obesity (BMI Z-score > 2)	2682(14.95)
Overweight (1 ≤ BMI Z-score ≤ 2)	3218 (17.94)
Normal weight (−2 < BMI Z-score < 1) Emaciation (BMI Z-score ≤−2)	11,195(62.41) 847(4.72)
Lipid profile	
Total cholesterol (mmol/L)	3.87 ± 0.73
Triacylglycerol (mmol/L) High-density lipoprotein (mmol/L)	0.76 ± 0.45 1.48 ± 0.35
Dyslipidemia^a^	1005 (31.22)

a. Dyslipidemia was defined as patients with total cholesterol > 5.17 mmol/L, triglycerides > 1.12 mmol/L, or HDL-C < 1.03 mmol/L.

Table 2 shows the comparison of BMI Z-score and BMI Z-score group between the fracture sites. The results showed that BMI Z-score and BMI Z-score group were statistically significant differences between different fracture sites (*P* < 0.001). After post hoc comparison, we found that BMI Z-score was statistically significant difference between every two groups (*P* < 0.05). Although no significant differences were observed in obesity between the upper limb and axial fracture groups Obesity was statistically significant difference between lower limb and head fracture groups (*P* < 0.05). This indicated that children with lower limb fractures (0.58 ± 1.74) exhibited the highest BMI Z-score, followed by those with upper limb fractures (0.50 ± 1.52), axial fractures (0.31 ± 1.56), and those with head fractures (−0.02 ± 1.52) had the lowest BMI Z-score. In terms of obesity prevalence, children with lower limb fractures exhibited the highest obesity rate (20.20%), whereas those with upper limb fractures (15.61%) and axial fractures (14.96%) displayed comparable obesity rates. Children with head fractures had the lowest obesity rate (8.42%). In terms of overweight prevalence, children with lower limb fractures (20.36%), upper limb fractures (18.54%) and axial fractures (17.22%) displayed comparable rates while children with head fractures had the lower overweight rate (14.27%). When it came to normal weight group, children with head fractures had the highest normal weight rate (69.96%), whereas those with upper limb fractures (62.55%) and axial fractures (61.05%) displayed comparable normal weight rates. Children with lower limb fractures had the lowest normal weight rate (53.81%). As for emaciation group, children with head fractures had the highest emaciation rate (7.35%), whereas those with lower limb fractures (5.63%) and axial fractures (6.77%) displayed comparable emaciation rates. Children with upper limb fractures had the lowest emaciation rate (3.31%).

**Table 2:** Comparison of BMI Z-score and obesity between fracture sites.

	Upper limb (*N* = 10175)	Lower limb (*N* = 3267)	Axial (*N* = 842)	Head (*N* = 3658)	F/χ^2^	*P*
BMI Z-score	0.50 ± 1.52	0.58 ± 1.74	0.31 ± 1.56	−0.02 ± 1.52	116.52	**< 0.001**
Obesity (BMI Z-score > 2)^a^	1588(15.61%)	660(20.20%)	126(14.96%)	308(8.42%)	197.03	**< 0.001**
Overweight (1 ≤ BMI Z-score ≤ 2)^b^	1886(18.54%)	665(20.36%)	145(17.22%)	522(14.27%)	49.17	**< 0.001**
Normal weight (−2 < BMI Z-score < 1)^c^	6364(62.55%)	1758(53.81%)	514(61.05%)	2559(69.96%)	192.49	**< 0.001**
Emaciation (BMI Z-score ≤−2)^d^	337(3.31%)	184(5.63%)	57(6.77%)	269(7.35%)	115.16	**< 0.001**

a. The difference in obesity rates between upper limb fractures group and axial fractures group showed no statistical significance (*P* > 0.05). b. The difference in overweight rates between upper limb fractures group, lower limb fracture and axial fractures group showed no statistical significance (*P* > 0.05). c. The difference in normal weight rates between upper limb fractures group and axial fractures group showed no statistical significance (*P* > 0.05). d. The difference in emaciation rates between lower limb fractures group and axial fractures group showed no statistical significance (*P* > 0.05).

In the healthy group, a total of 3219 individuals were included. Among them, 1955 (60.73%) were male and 1264 (39.27%) were female. The mean age of the participants was 4.97 ± 3.50 years. The more details are shown in Table 3.

**Table 3:** Distribution of children’s characteristics in the healthy group.

Variables (*N* = 3219)	Mean ± SD/*n* (%)
Age	4.97 ± 3.50
Gender	
Male	1955 (60.73)
Female	1264 (39.27)
BMI Z-score	0.15 ± 1.43
Obesity (BMI Z-score > 2)	277(8.61)
Overweight (1 ≤ BMI Z-score ≤ 2)	483(15.00)
Normal weight (−2 < BMI Z-score < 1) Emaciation (BMI Z-score ≤−2)	2349(72.97) 110(3.42)
Lipid profile	
Total cholesterol (mmol/L)	4.13 ± 0.79
Triacylglycerol (mmol/L)	0.94 ± 0.54
High-density lipoprotein (mmol/L)	1.50 ± 0.32
Dyslipidemia^a^	794 (24.67)

a. Dyslipidemia was defined as patients with total cholesterol > 5.17 mmol/L, triglycerides > 1.12 mmol/L, or HDL-C < 1.03 mmol/L.

Due to variations in gender and age baselines, we employed a 1:1 Propensity Score Matching to match the fracture and healthy groups based on gender and age. Finally, we got a new pair of 3219 children in the fracture and healthy groups. Table 4 the comparison of physiological parameters between the fracture and healthy groups. We found statistically significant differences in BMI Z-score (*P* < 0.001), obesity (*P* < 0.001), and between the fracture and healthy groups, along with the lipid profile such as total cholesterol, triacylglycerol, high density lipoprotein and dyslipidemia (*P* < 0.001).

**Table 4:** Comparison of BMI Z-score, obesity and lipid profiles between the fracture and healthy groups after PSM.

Variables	Fracture(*N* = 3219)	Healthy(*N* = 3219)	t/χ^2^	*P*
BMI Z-score	2.43 ± 1.22	0.15 ± 1.43	37.56	**< 0.001**
Obesity	2027(62.97%)	277(8.61%)	2070.02	**< 0.001**
Lipid profile				
Total cholesterol (mmol/L)	3.91 ± 0.74	4.13 ± 0.78	−11.58	**< 0.001**
Triacylglycerol (mmol/L)	0.89 ± 0.52	0.94 ± 0.54	−3.70	**< 0.001**
High-density lipoprotein (mmol/L)	1.37 ± 0.33	1.50 ± 0.32	−15.93	**< 0.001**
Dyslipidemia^a^	1005 (31.22%)	794 (24.67%)	34.345	**< 0.001**

a. Dyslipidemia was defined as patients with total cholesterol > 5.17 mmol/L, triglycerides > 1.12 mmol/L, or HDL-C < 1.03 mmol/L.

We further compare the difference in the lipid profile between the fracture and healthy group among children with obesity in Table 5. The results showed that there were significant differences in total cholesterol, and triacylglycerol between the fracture and healthy groups (*P* < 0.001).

**Table 5:** Comparison of lipid profile between the fracture and healthy groups among children with obesity after PSM.

Variables	Fracture(*N* = 2027)	Healthy(*N* = 277)	t/χ^2^	*P*
Total cholesterol (mmol/L)	3.93 ± 0.74	4.11 ± 0.71	3.68	**< 0.001**
Triacylglycerol (mmol/L)	0.92 ± 0.56	1.21 ± 0.91	5.23	**< 0.001**
High-density lipoprotein (mmol/L)	1.36 ± 0.32	1.38 ± 0.30	0.83	**0.407**
Dyslipidemia^a^	752(32.64%)	103(37.18%)	2.959	0.085

a. Dyslipidemia was defined as patients with total cholesterol > 5.17 mmol/L, triglycerides > 1.12 mmol/L, or HDL-C < 1.03 mmol/L.

To evaluate the association between obesity and the odds of fractures, we fit a logistic regression model between BMI Z-score and fracture sites in Table 6. BMI Z-score showed a statistically significant positive association with fracture (*P* < 0.001, OR = 4.885, 95%CI: 4.53–5.27) in unadjusted model, which indicated that a 1-unit increase in BMI Z-score is associated with a 4.8-fold higher odds of fracture risk. In model adjusted by age and gender, BMI Z-score showed a statistically significant positive association with fracture (*P* < 0.001, OR = 6.568, 95%CI: 5.986–7.207). Significant positive statistical differences were observed among all fracture sites.

**Table 6:** Association between BMI Z-score and various fracture sites.

Fracture Sites	Model 1^a^		Model 2^b^	
	OR(95% CI)	*P*	OR(95% CI)	*P*
All fracture	4.885(4.533–5.265)	< 0.001	6.568(5.986–7.207)	< 0.001
Upper limb	5.652(5.198–6.144)	< 0.001	7.143(6.468–7.887)	< 0.001
Lower limb	5.788(5.268–6.360)	< 0.001	7.210(6.442–8.069)	< 0.001
Axial	5.075(4.244–6.069)	< 0.001	5.980(4.835–7.396)	< 0.001
Head	3.444(3.147–3.770)	< 0.001	5.666(5.060–6.344)	< 0.001

a. Unadjusted model; b. Model adjusted by gender and age.

## Discussion

In this study, we explored the differences in BMI Z-score and obesity in various fracture sites and then investigated the relationship between obesity and odds of fracture. We found that children with lower limb fractures exhibited the highest BMI Z-score, followed by those with upper limb fractures and axial fractures, and those with head fractures had the lowest BMI Z-score. In addition, children with lower limb fractures exhibited the highest obesity rate, whereas those with upper limb fractures and axial fractures displayed comparable obesity rates. While those with head fractures had the lowest obesity rate. When comparing children between the fracture and healthy groups, the result indicated that there were significant differences in BMI Z-score, obesity, and dyslipidemia between the fracture and healthy groups. Employing logistic models, we explored the associations between BMI Z-score and odds of fracture. The result indicated that the BMI Z-score was associated with an increased risk of fractures.

Several authors have noted the increased risk of fracture, especially lower limb fracture in obese patients. Research in the USA reported a greater prevalence of fractures in overweight children and anatomic tibiofemoral angle measurements showed greater malalignment in overweight compared with normal weight children^17^. Haricharan et al. indicated that obese adolescents involved in motor vehicle collisions had an increased risk of severe lower limb injuries but decreased risk of head injuries^18^. A study conducted in Spain on preschool obese children indicated that children with an overweight and obese range BMI had an increased risk of lower limb fracture and upper limb fracture^5^. A study in the USA also reported the association between childhood obesity and increased risk of most lower limb fractures^19^. Taken together, these studies are consistent with our results.

The impact of obesity on fractures has always been considered to have a bidirectional effect. Previous studies found that children and adolescents with obesity have higher bone mass, bone dimensions, and bone mineral density than normal-weight peers, indicating that the adipose tissue exerts a positive effect on bone structure^20–22^. Although it has been hypothesized that the cushion effect of excess body fat can also be protective^6^, many studies have reported that obesity influences the bone microenvironment and health by various mechanisms. In a mice model of reduced hepatic insulin clearance, hyperinsulinemia, which can be seen in some obese children, has been associated with reduced bone turnover and, consequently, poor bone quality^7^. A study on male mice also reported that obesity may modify the bone marrow microenvironment and direct bone marrow mesenchymal cells to adipogenesis, which reduces bone formation and increases bone marrow adipose tissue^8^. A study using high-resolution peripheral quantitative computed tomography indicated that a reduction in bone strength may occur in the tibia of obese children reflecting a negative impact of fat mass on bone strength in weight-bearing sites^23^. A biomechanical study on arm fractures in obese boys indicated that even though the thicker fat layer has a better cushion effect, the greater momentum effect of the body mass is dominant over the cushion effect^24^. Another study indicated the low-grade inflammation that obesity may bring would also influence endochondral longitudinal bone growth together with changes in nutrients, minerals, and hormone metabolism which may lead to an increased risk of fracture^9^. Moreover, obesity would alter levels of numerous molecules, such as TNFα, DKK1, sclerostin, IL-6, serotonin, and advanced glycation end products, which could inhibit osteoblastogenesis^25^.

Besides these physiological and pathological mechanisms, obesity is often associated with unhealthy lifestyle habits. Multiple articles have demonstrated that most obese children always had low levels of physical activity compared with nonobese counterparts^17,26–28^ and physical activity can be a major mechanical stimulus for bone accretion^29^. Increased body mass may increase the impact during a fall, decrease protective response, and poor balance may increase the risk of falling. These can all contribute to the increased fracture risk seen in obese children^26^.

Additionally, this study found that the fracture group had lower mean total cholesterol levels and triacylglycerol levels compared to the healthy group, but showed lower HDL levels and a higher prevalence of dyslipidemia. This seems to suggest that blood lipids may have a bidirectional effect on fracture risk. As there were few studies discussing the relationship between childhood fractures and blood lipids, we reviewed numerous findings from adult population studies and found the results of these studies highly controversial. Numerous studies have found the association between blood lipids and fracture risk. A study conducted in the adult population of Sweden suggested that elevated total cholesterol levels were associated with the long-term risk of fractures^30^. Additionally, a study among elderly diabetic individuals in Beijing indicated that higher HDL levels served as a protective factor against fractures^31^. A multi-ethnic female cohort study in the United States revealed a positive correlation between total triacylglycerol levels and fracture risk^32^. However, we also found numerous researches suggesting no association or even negative correlation between blood lipids and fracture risk. A meta-analysis showed that individuals with low HDL levels had a lower fracture risk compared to those with normal HDL levels^33^. Additionally, a study involving 958 postmenopausal Korean women indicated that vertebral fracture patients had lower TC and TG levels than those without vertebral fractures^34^. These discrepancies may be attributed to the distinct effects of different lipid types on bone health. A study based on the UK Biobank showed that unsaturated fatty acids and docosahexaenoic acid (DHA) increased fracture risk in males, whereas high levels of HDL, 3-hydroxybutyrate, and sphingomyelin reduced fracture risk in females^10^. However, our study only measured overall blood lipid profiles without specifying each lipid type, indicating that further research is needed in this regard.

Our study also found that head fractures are less common in obese children. This result can be explained from two aspects. Firstly, obese children have thicker fat on their heads, which provides some protective effect. Secondly, it may be due to the reduced fracture risk caused by less physical activity among obese children. Surveys from different regions around the world have shown that the main causes of head injuries in children are traffic accidents and sports injuries, which may explain the low incidence of head fractures in obese children^35–37^.

In light of our study findings, we recommend that obese children should implement suitable weight management strategies to mitigate the risk of fractures. When physical exercise is chosen as a weight - loss approach, additional safety precautions should be strictly adhered to in order to prevent potential injuries especially on lower limbs. Moreover, for pediatric patients presenting with trauma but lacking evident clinical manifestations of fractures, BMI and dyslipidemia status should be integrated into the fracture risk assessment protocol. This integration will facilitate the determination of whether further fracture-related diagnostic screenings are warranted.

Our research presents several notable strengths. Firstly, to our knowledge, this study is the first to explore the differences in BMI Z-score and obesity in different fracture sites. Previous studies have always focused on only one or two fracture sites. Secondly, unlike many previous studies that only concentrate on fractured children, we introduced healthy children to analyze the association between obesity and fractures.

While our study exhibits notable strengths, it also harbors certain limitations. Firstly, we only included children who were diagnosed with fractures for the first time and we therefore did not take into account refractures. This may decrease the number of overall fractures for the group as a whole. Secondly, our sample size of the healthy group was relatively modest compared with the fracture group. This may lead to statistical bias. As a countermeasure, we have used propensity score matching to minimize statistical bias as much as possible. Thirdly, the study was conducted at a single center, limiting the generalizability of our findings to broader populations beyond Nanjing. Consequently, caution should be exercised in extrapolating our results to other regions or demographic groups. For further study, we should take the next step to measure bone mineral density for analyzing the relationship between blood lipids, bone density, and fractures. In addition, the biological samples of both groups should be collected to analyze lipid metabolism. Finally, further evidence from larger, multi-center studies is needed.

## Conclusion

Our findings suggest that children with lower limb fractures exhibited the highest BMI Z-score and obesity rate, while those with head fractures had the lowest BMI Z-score and obesity rate. When compared with the healthy group, fractured children had higher BMI Z-score, obesity, and dyslipidemia rates. BMI Z-score was associated with an increased risk of fractures. In summary, weight management strategies are recommended for obese children to mitigate fracture risk, especially when exercise is utilized for weight reduction, where stringent safety protocols should be implemented. Additionally, for pediatric patients who present with trauma but exhibit no overt signs of fractures, both BMI and dyslipidemia status should be integrated into the fracture risk assessment framework to inform appropriate screening decisions.

## Electronic supplementary material

Below is the link to the electronic supplementary material.

Supplementary Material 1

## Data Availability

The datasets generated during and/or analysed during the current study are available from the corresponding author on reasonable request.
