# Supplementary material for: The impact of childhood obesity on different fracture sites
Source: Sci Rep. 2025 Jul 20;15:26338. doi: 10.1038/s41598-025-11203-7 (PMC12277405; doi:10.1038/s41598-025-11203-7)
Supplement: Supplementary file 1 — Supplementary Material 1 [file 41598_2025_11203_MOESM1_ESM.docx]

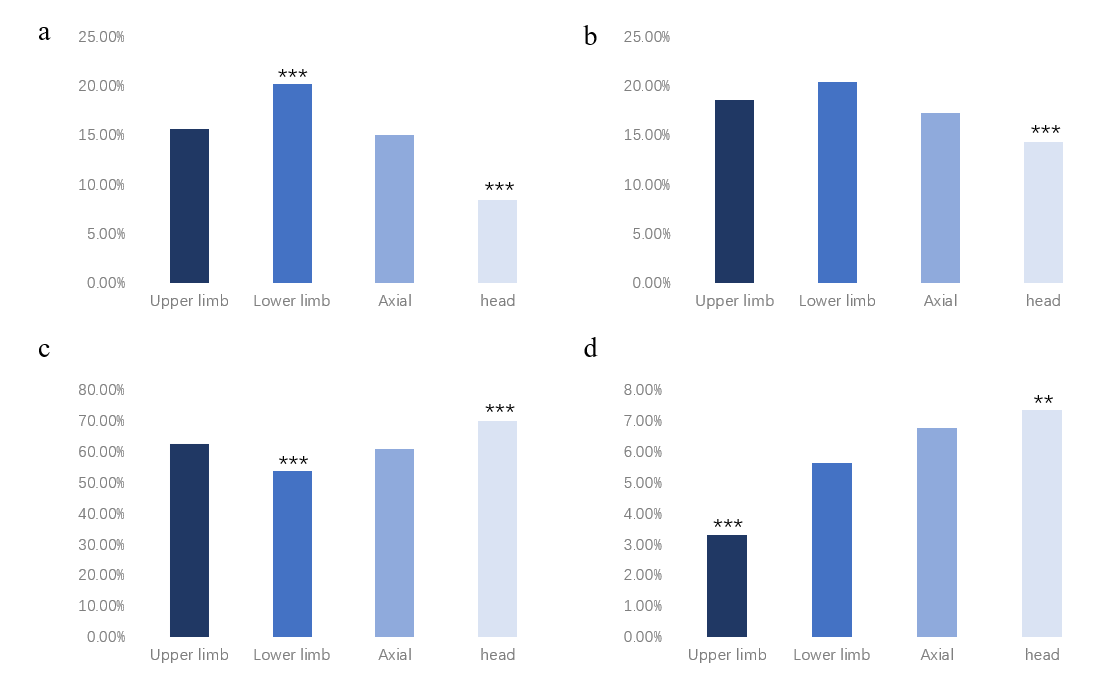


Supplementary Figure 1 Comparison of BMI Z-score group in different fracture sites

a. Obesity b.Overweight c.Normal weight d.Emaciation
